# Systemic corticosteroid dose–response effects in asthma: an observational cohort study

**DOI:** 10.1183/23120541.00172-2024

**Published:** 2025-01-27

**Authors:** Xiao Xu, Trung N. Tran, Sarowar Golam, Victoria Carter, David B. Price

**Affiliations:** 1 AstraZeneca, Gaithersburg, MD, USA; 2 AstraZeneca, Gothenburg, Sweden; 3 Observational and Pragmatic Research Institute, Singapore, Singapore; 4 Division of Applied Health Sciences, Centre of Academic Primary Care, University of Aberdeen, Aberdeen, UK

## Abstract

Systemic corticosteroids (SCS) are prescribed to manage acute exacerbations of asthma, as recommended by all national and international guidelines for treatment of asthma. However, cumulative SCS exposure is associated with adverse outcomes, higher healthcare resource utilisation and higher associated costs [1–3], often in a dose-dependent association [4]. There is a call to action to balance the benefits and harms of SCS in asthma from the World Allergy Organization and the Respiratory Effectiveness Group [5]. Furthermore, there is established evidence that SCS use is strongly associated with increased all-cause mortality and cardiovascular mortality in a range of immune-mediated inflammatory diseases, such as rheumatoid arthritis, polymyalgia rheumatica, giant cell arteritis and inflammatory bowel disease [6, 7]. However, the association between SCS use and mortality in asthma has not been fully characterised [8].

## Shareable abstract

**This study is among the first in a large patient database over an extended period to identify a link between SCS use/overuse and mortality in asthma in a positive dose–response relationship with average daily exposure and cumulative dose categories** https://bit.ly/3zzl2QN

*To the Editor:*

Systemic corticosteroids (SCS) are prescribed to manage acute exacerbations of asthma, as recommended by all national and international guidelines for treatment of asthma. However, cumulative SCS exposure is associated with adverse outcomes, higher healthcare resource utilisation and higher associated costs [1–3], often in a dose-dependent association [4]. There is a call to action to balance the benefits and harms of SCS in asthma from the World Allergy Organization and the Respiratory Effectiveness Group [5]. Furthermore, there is established evidence that SCS use is strongly associated with increased all-cause mortality and cardiovascular mortality in a range of immune-mediated inflammatory diseases, such as rheumatoid arthritis, polymyalgia rheumatica, giant cell arteritis and inflammatory bowel disease [6, 7]. However, the association between SCS use and mortality in asthma has not been fully characterised [8]. Despite the use of add-on treatments to inhaled corticosteroids and biologics, use and overuse of long-term oral corticosteroids (OCS) remain common [9]. Prior associations between SCS use and mortality maybe due to SCS treatment alone or confounded by severity [4, 10]. Recently, Danish investigators reported an association of OCS with asthma mortality even with low cumulative doses ≤500 mg prednisolone-equivalent, and with a dose response [11]. These results, however, were limited by the lack of diagnostic data from primary care on some comorbidities, no continuous SCS, time-dependent assessment for regression models and other study design issues (only patients aged 18–45 years old, the follow-up duration of SCS nonusers was significantly shorter than that of SCS users, *etc.*) [12]. We aimed to identify dose–response associations between SCS exposure and all-cause mortality and mortality from SCS-associated adverse events in a large population of patients with asthma over an extended period.

We conducted an observational, historical, cohort study using the Clinical Practice Research Datalink (CPRD) in the UK. Data from June 1994 to January 2015 were drawn from the CPRD database, and linked with 2016 Hospital Episode Statistics (HES) and mortality data from the Office of National Statistics (ONS) [2]. This research followed and endorsed the STROBE guidance for reporting observational research [13]. The protocol was approved by the CPRD Independent Scientific Advisory Committee (17_002; ENCEPP EUPAS15175). Eligible patients were adults (18 years and older) with asthma, defined as physician-diagnosed and a validated diagnostic code recorded for asthma (with no subsequent code for asthma resolved); no SCS-related adverse events prior to the index date, or adrenal insufficiency or Addison's disease ever, and no diagnosis of cancer 5 years before or 3 months after the index date; no tamoxifen prescription for breast cancer; and ≥3 years of continuous practice records (≥1 year of baseline and ≥2 years of follow up), with no prior evidence of SCS use before the index period, and two or more respiratory drug prescriptions in the year before and/or after SCS initiation) [1, 2]. We evaluated SCS exposure-related mortality associated with adverse outcomes or with incident morbidities that may be SCS-related, such as diabetes, osteoporosis and cardiovascular disease. Global Initiative for Asthma (GINA) 2021 asthma severity was assessed with the highest step during the baseline and outcome years, and with the daily dosage of drugs based on the last prescription in each period, as described elsewhere [14]; those with insufficient data were labelled as “GINA 0”, as we did not performed any imputation on missing data. For statistical analyses, the incidence rates (IR) of all-cause mortality were compared using the IR difference and the IR ratio with their 95% confidence intervals. Cumulative dose in grams of prednisolone-equivalent SCS with conversion factors 0.16 for dexamethasone, 0.4 for hydrocortisone and 0.8 for methylprednisolone, and average daily exposure (milligrams per day) were used as continuous, time-dependent variables for regression models. A time-to-event design and multivariable Cox proportional hazard models were used.

In total, from an original population of >5 million subjects, 16 623 asthma patients were identified as per our inclusion and exclusion criteria, and finally there were 9413 asthma patients with linked HES and ONS data. Mean±sd age was 49.0±17.0 years, and there were 3268 (34.7%) men and 6145 (65.3%) women; their smoking status was 4592 (49.2%) non-, 2861 (30.7%) current and 1877 (20.1%) ex-smokers, while in 83 (0.9%%), there were no data. Overall, GINA 2021 severity distribution was 1688 (17.9%) step 1, 2531 (26.9%) step 2, 1846 (19.6%) step 3 and 1547 (16.4%) step 4, while 1801 (19.1%) were GINA 0. The mean follow-up period was 9 years, ranging from 2 to 29 years, and all GINA control and severity stages were represented. Overall, a total of 1762 deaths were observed. Respiratory tract disease was the most frequent primary cause of death (30%) and cardiovascular disease was the second (27%). Risk of death started to increase sharply after year 8. There was a positive dose–response association between cumulative and average daily SCS exposure and all-cause mortality. Cumulative death was greater with longer duration of follow-up within each dose group, and more importantly, with higher rates observed in patients with greater SCS exposure levels. Patients with SCS daily exposure ≥5 mg·day^−1^ experienced the greatest risk of death. Similar results were observed within risk cohorts for selected (based on prior research) incident SCS-related morbidities for type-2 diabetes, osteoporosis and cardiovascular disease. In multivariable analyses, patients exposed to a cumulative prednisolone-equivalent SCS dose ≥10 g had a greater than two-fold risk of death compared with patients exposed to the lowest cumulative dose, <0.5 g (hazard ratio (95% CI) 2.17 (1.78–2.64)) (figure 1). Patients with an average daily SCS exposure ≥7.5 mg·day^−1^ were 4.6 times more likely to die (hazard ratio (HR) (95% CI) 4.56 (3.45–6.04)), whereas those with an average daily exposure from 0.5 to <2.5 mg·day^−1^ were 1.5 times more likely to die (1.52 (1.37–1.69)), compared with patients who had <0.5 mg·day^−1^ average daily dose.

**FIGURE 1:**
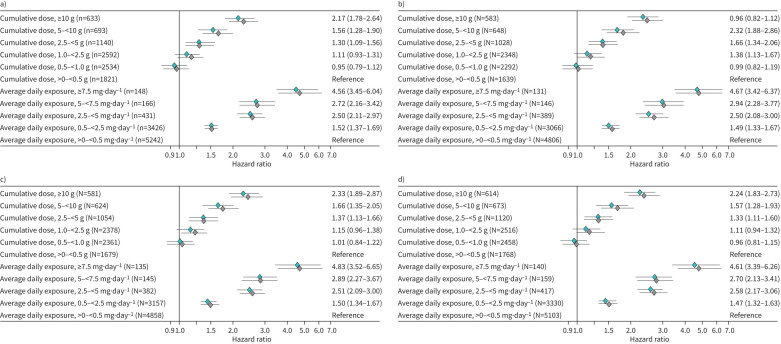
Adjusted model results for risk of death associated with cumulative dose and average daily exposure of systemic corticostroids for a) all patients, and the b) cardiocerebrovascular disease, c) type 2 diabetes mellitus and d) osteoporosis risk cohorts. Grey indicates unadjusted and green indicates adjusted hazard ratios. Cumulative dose exposure strata go up to ≥10 g prednisolone-equivalent or greater, with those asthmatic patients exposed to <0.5 g considered as the reference category; and average daily exposure strata go up to ≥7.5 mg·day^−1^ prednisolone-equivalent with those asthmatic patients exposed to <0.5 mg·day^−1^ considered as the reference category.

All previous studies compared long-term OCS use *versus* intermittent or non-OCS use, or with OCS status categorised at baseline only, whereas we used conditional multivariable Cox proportional hazard models with time-to-event design and adjustments for age, sex and other confounders (smoking status, body mass index, number of antibiotic-treated infections in the last year and comorbidities (hypertension, depression, peptic ulcer, dyslipidaemia, type 2 diabetes mellitus and cataracts)) using an automated confounder selection approach, exploring outcomes in asthma patients with incremental SCS exposure for up to 29 years.

Our study has a number of limitations. There must be caution when exploring causation, and potential confounding by severity may explain part of the associations [15]. Beyond any effort to tackle confounding by severity, use of SCS may simply reflect more severe asthma, which may be associated with increased comorbidities, such as cancer and cardiovascular disease, and else independent of SCS use, which may at least in part just reflect more severe asthma. We endorse the STROBE guidance [13] but the observational nature of our research cannot rule out indication/severity bias as beyond randomised controlled trials; a requirement of SCS is a well-established determinant of asthma severity and mortality. Our results do not apply to children. Furthermore, given the sequential exclusion of those with shorter follow-up, no respiratory prescriptions during our defined assessment period, and no HES or ONS linkage, the generalisability of our results requires further testing. A final limitation is that we did not explore the potential effect modification by performing sensitivity analyses according to GINA asthma severity classification. GINA 2023 recommends that prolonged maintenance SCS treatment should be avoided, and instead biologics and other therapies should be the preferred add-on treatments. Furthermore, strategies to minimise SCS use should be prioritised [9], all of which are endorsed by the World Allergy Organization and the Respiratory Effectiveness Group. Our study showed that increasing cumulative SCS exposure (≥1 g, or four or more courses of SCS in a lifetime) and increasing mean daily SCS exposure (≥2.5 mg·day^−1^) places asthma patients at a greater risk of mortality, either for all-cause or SCS-related adverse outcomes. These findings highlight the importance of improving the awareness of SCS-related adverse effects and mortality. It also supports recommendations from GINA and others that patients with repeated exacerbations requiring several SCS courses should be thoroughly assessed in primary care or referred to a hospital specialist to examine alternative strategies [9, 16], with many not currently referred or discharged back to primary care with substantial use [17]. Furthermore, it supports the need for continuing development and identification of alternative treatments for treating asthma to reduce adverse clinical events and sparing SCS use (or dose).

We conclude that risk of death among asthma patients treated with SCS was associated in a positive dose–response relationship with average daily exposure and cumulative dose categories. This study is among the first to examine the link between SCS use/overuse and mortality in asthma in a large patient database over an extended period. These findings have important clinical implications: healthcare providers may want to evaluate other treatment options and strategies for patients with asthma who are using repeated courses of SCS, targeting reduction in the risk of mortality and steroid-related adverse events.
